# Modality and terminology changes for behavioral health service delivery during the COVID-19 pandemic: a systematic review

**DOI:** 10.3389/fpsyt.2023.1265087

**Published:** 2024-02-05

**Authors:** Kimberly S. Elliott, Eman H. Nabulsi, Nicholas Sims-Rhodes, Vandy Dubre, Emily Barena, Nelly Yuen, Michael Morris, Sarah M. Sass, Bridget Kennedy, Karan P. Singh

**Affiliations:** ^1^Department of Healthcare Policy, Economics and Management, University of Texas at Tyler Health Science Center, Tyler, TX, United States; ^2^Department of Epidemiology and Biostatistics, University of Texas at Tyler, Tyler, TX, United States; ^3^Robert R. Muntz Library, The University of Texas at Tyler, Tyler, TX, United States; ^4^Department of Psychology and Counseling, The University of Texas at Tyler, Tyler, TX, United States

**Keywords:** behavioral health, behavioral health modality, COVID-19, pandemic, telehealth, telemedicine

## Abstract

**Introduction:**

The COVID-19 pandemic prompted healthcare professionals to implement service delivery adaptations to remain in compliance with safety regulations. Though many adaptations in service delivery were reported throughout the literature, a wide variety of terminology and definitions were used.

**Methods:**

To address this, we conducted a PRISMA review to identify service delivery adaptations across behavioral healthcare services in the United States from March 2020 to May 2022 and to identify variations in terminology used to describe these adaptations. We identified 445 initial articles for our review across eight databases using predetermined keywords. Using a two-round screening process, authors used a team approach to identify the most appropriate articles for this review.

**Results:**

Our results suggested that a total of 14 different terms were used to describe service modality changes, with the most frequent term being *telehealth* (63%). Each term found in our review and the frequency of use across identified articles is described in detail.

**Discussion:**

Implications of this review such as understanding modality changes during the COVID-19 pandemic and beyond are discussed. Our findings illustrate the importance of standardizing terminology to enhance communication and understanding among professionals.

## 1 Introduction

The COVID-19 pandemic presented a unique challenge to healthcare delivery in the U.S. In an effort to provide continued care to patients while reducing the risk of virus transmission, healthcare institutions adapted their modes of service delivery in accordance with the Center for Disease Control’s (CDC) safety regulations and social distancing guidelines (1). During this time, there was a significant increase in the integration of technology-based health services and telecommunications. Although not a new process for many healthcare providers and institutions, the rapid shift necessitated a change in institutional and state level policies and procedures to help streamline the transition to a virtual care delivery model. Some of the immediate changes included the relaxing of Health Insurance Portability and Accountability Act (HIPAA) regulations by the U.S. Department of Health and Human Services (HHS) to allow for the use of various virtual conferencing platforms, as well as modifications in telehealth services covered by Medicaid/Medicare (2, 3). In addition, institutions expanded their IT infrastructure to create a pathway for online only operations and acquired additional technological equipment to utilize telehealth services (4).

Particularly for mental health services, the shift to remote/virtual service delivery was important for meeting the increased need for mental health services during the pandemic (5). Mental health providers reported an increased use of technology-based health services, with approximately 86% of the work of psychologists moved to virtual platforms and around 67% of psychologists conducting their clinical work via telepsychology (3). Virtual adaptations were made for various patient populations and varying degrees of complex clinical conditions, including trauma-focused care, treatment for individuals with intellectual disabilities, and individuals with serious mental illness (e.g., schizophrenia). Overall, these implementation changes ranged across provider and service type, as well as institutional setting. Understanding these adaptations is crucial for future preparedness and healthcare planning as it provides insights into effective strategies used which can inform healthcare systems for future crises.

Although institutions provided remote/virtual services through similar methods and platforms, they varied on which terms they used to describe their services and how they defined those terms. For example, “telehealth” and “telemedicine” were commonly used to discuss services offered via a virtual delivery model. However, how those terms were defined, and the types of practices encompassed within each term differed across settings (6). Moreover, multiple terms were used to describe the same practices and services, resulting in greater lexicon diversity.

The purpose of this PRISMA review was twofold: (1) to identify variations in the terminology and definitions used to describe such changes across healthcare settings and institutions and (2) to identify care modality changes implemented across healthcare systems and institutions in the United States during the COVID-19 pandemic (March 2020–May 2022). The expanded adoption of telehealth use provides an important opportunity for researchers and clinicians to further investigate the implementation and use of technology-based health services for patients. In addition, gaining a better understanding of terms and definitions for technology-based health services can help provide greater clarity around practices and services delivered, create more uniformity in language, and potentially impact the application of such services across healthcare systems.

## 2 Methods

### 2.1 Eligibility and search criteria

Studies were included in this review if they were written in English, studies focused in the United States on mental health modality changes due to Covid response, published after March 11, 2020. Search criteria included Covid OR pandemic AND Telehealth OR “behavioral health” OR psychotherapy OR counseling OR psychiatry OR “mental health care” OR “health care delivery” both in the abstract with limiters of scholarly Peer Reviewed Journals. The full text only searching criteria option was not used as it allowed for discovery of the best articles for the study. Full text articles not recovered were interlibrary loaned from other universities.

### 2.2 Information sources

To meet the research needs of this study, eight databases were individually searched to identify the articles for review: APA PsycArticles, APA PsycINFO, Ebscohost Psychology & Behavioral Sciences Collection, PubMed, Scopus, CINAHL, Proquest Central, and Web of Science. These databases were selected as the key databases for psychological research available to the searcher as well as presenting a wide range of journals. Potential search limitations include dates of searching and the limitation of location. Multinational studies that included the United States were excluded.

### 2.3 Initial study selection

The review was guided by the Preferred Reporting Items for Systematic Reviews and Meta-Analysis (PRISMA) 2020 methodology and matrix. The initial database search was conducted by a university research librarian. Titles, article metadata including keywords, abstracts, and geographical location of the study were scanned to identify appropriate articles. The initial search resulted in 445 articles. Utilizing the Sciwheel reference management system, the university accessible databases, and interlibrary loan, the full text of all identified articles was accessed using a collective process.

**Figure 1:**
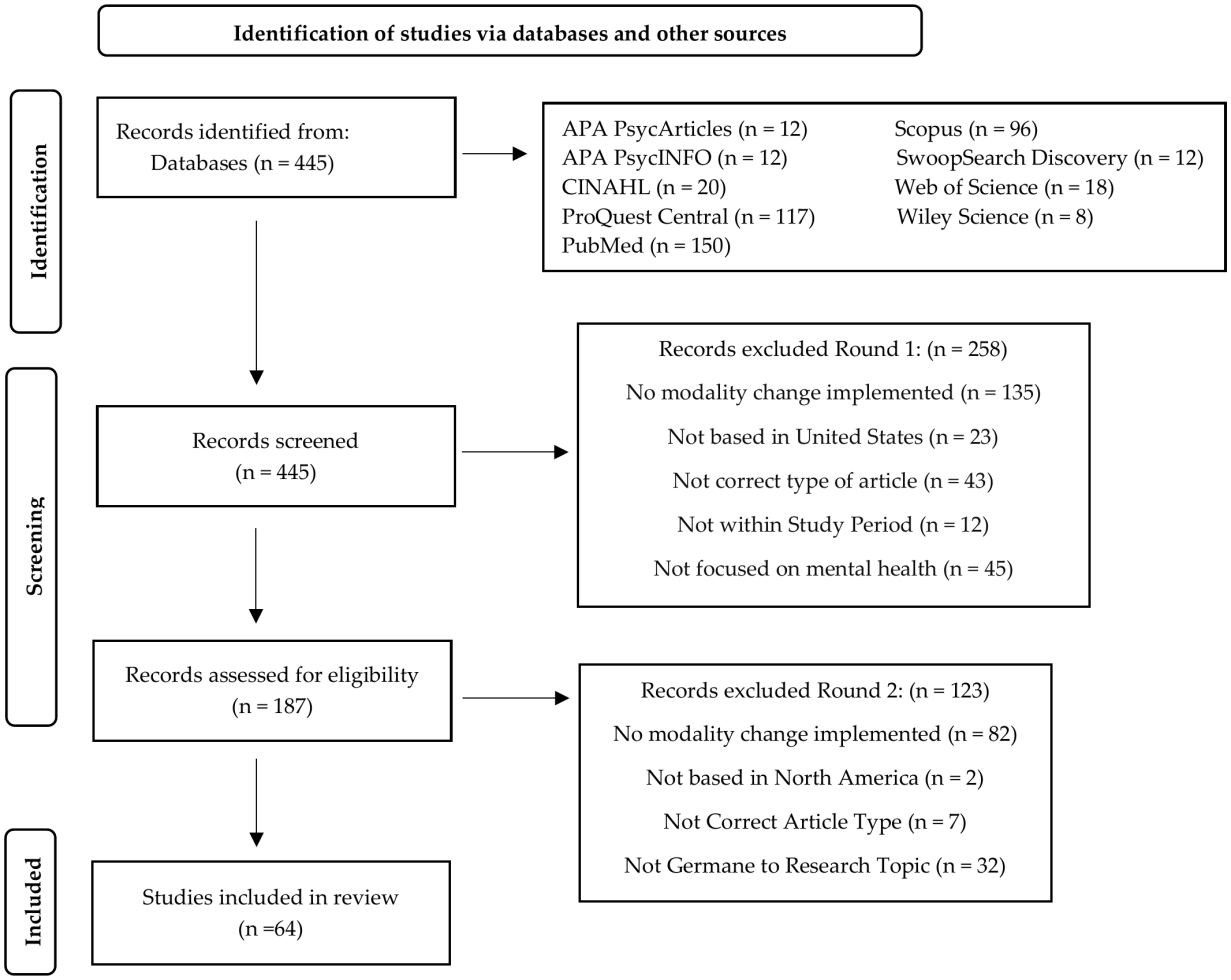
Preferred reporting items for rapid reviews and meta-analysis (PRISMA) figure that demonstrates the study selection process.

Through the Microsoft Teams platform, virtual meetings were held, and data and files were collected and organized. Excel was used for data collection and to categorize and rate each article regarding inclusion criterion. A two-round screening process was used for exclusion screening to identify the most appropriate articles for this review, to eliminate bias, and eliminate reader fatigue. An affinity matrix was used by all researchers to identify and codify themes within each article. Figure 1 provides the schematic flow of the sample identification and selection process.

## 3 Results

A total of 14 terms were used to describe how behavioral health care was delivered as a result of the pandemic. Table 1 shows the frequency of behavioral health modalities used as a result of the restrictions imposed by the COVID-19 pandemic.

**Table 1:** Modality occurrence frequencies and corresponding articles.

Modality	Occurrence frequency
Telehealth	63%
Telemedicine	23%
Telemental health	17%
Telepsychiatry	11%
Teletherapy	9%
Telebehavioral health	6%
Telepsychology	3%
Virtual delivery	2%
Videoconferencing	2%
Telephone-based psychotherapy	2%
Teleconsultation	2%
Video telehealth	2%
Digital health	2%
Unguided mental health program	2%

Delivery modalities can be categorized as either audio-only or audio-visual. The modes of delivery were synchronous, asynchronous, or hybrid combinations. There were only two instances where the modalities described were delivered online without audio or audiovisual platforms. These two exceptions involved the use of online web or app-based programs. Table 2 shows the total sample selected and gives an overview of the sample data.

**Table 2:** Study sample article title, authors and a summary of findings.

Article title	Authors	Findings/Summary
Eliciting emotional expressions in psychodynamic psychotherapies using telehealth: a clinical review and single case study using emotional awareness and expression therapy	Ahlquist and Yarns (7)	This article discusses the implementation of psychodynamic therapy via telehealth and describes clinical experiences transitioning a psychodynamically-informed, evidence-based, and experiential group treatment for chronic pain, emotional awareness, and expression therapy (EAET), to video telehealth at VA Greater Los Angeles Healthcare System. The authors reported numerous challenges in delivering psychodynamically-informed care via telehealth, including difficulty establishing and maintaining eye contact, difficulty hearing patients and seeing patients’ full bodily emotional expressions, patients dropping off and on the Zoom chat, interruptions due to phones ringing or other noises in the homes of the patients, and even patients getting up and walking away from the camera during the session.
Implementing COVID19 Mitigation in the Community Mental Health Setting: March 2020 and Lessons Learned	Alavi et al. (8)	This article details the development of patient prioritization procedural documents during the COVID-19 pandemic by physicians and non-physicians across state-funded community mental health service programs (CMHSP) in Michigan. The authors specifically discuss one region’s response and outline the processes which allowed continuity of care during the pandemic.
Adaptation of an academic inpatient consultation-liaison psychiatry service during the SARS-CoV-2 pandemic: Effects on clinical practice and trainee supervision	Beran and Sowa (9)	The present article describes implementation changes to a virtual care delivery model in a large, academic psychiatry consult liaison service during the pandemic. Authors reported a lower consultation volume and average weekly charges compared with pre-pandemic volumes. Trainees and attendings reported overall moderate satisfaction with telephone and video consultations and learning experiences.
Adaptations made to pediatric consultation-liaison psychiatry service delivery during the early months of the COVID-19 pandemic: A North American multisite survey	Brahmbhatt et al. (10)	This article describes early implementation changes by 22 pediatric consultation-liaison psychiatry service providers during the COVID-19 pandemic. Authors reported on COVID-related changes to hospital practices and procedures including restricted visitation to one caregiver, use of virtual rounding, ongoing trainee involvement, and an overall low number of COVID-positive pediatric patients.
Disruptions in care for Medicare beneficiaries with severe mental illness during the COVID-19 pandemic	Busch et al. (11)	The present article discusses disruptions in care for 650,000 Medicare beneficiaries with serious mental illness, including schizophrenia and bipolar I disorder. Authors found significant decreases in mental health–related outpatient, emergency department, and inpatient use as well as medication fills, particularly early in the pandemic, despite widespread use of telemedicine. Furthermore, they reported a significant decrease in outpatient utilization among individuals who were Black, had dual Medicare-Medicaid eligibility, or had disabilities.
Availability of outpatient telemental health services in the United States at the outset of the COVID-19 pandemic	Cantor et al. (12)	This study examines the availability of telehealth services at outpatient mental health treatment facilities in the United States at the outset of the COVID-19 pandemic. Results found that approximately 43% of outpatient mental health facilities in the United States reported telehealth availability at the outset of the pandemic. The authors reported that facilities located in the southern U.S. and nonmetropolitan counties, facilities with public sector ownership, those providing care for both children and adults, and those accepting Medicaid as a form of payment were more likely to offer telehealth services. By contrast, outpatient mental health treatment facilities located in states with state-wide shelter-in-place laws were less likely to offer telehealth, as well as facilities in counties with more COVID-19 cases per 10,000 population.
Development of a virtual consultation-liaison psychiatry service: a multifaceted transformation	Caravella et al. (13)	This article discusses changes to a consultation-liaison (CL) psychiatry service at NYU Langone Health Tisch/Kimmel/Orthopedic Hospital to a primarily virtual CL service. Some challenges identified by the authors included the modification of workflows to address the individual needs of each clinical setting. The article further discusses each setting’s existing telemedicine capabilities and resources, ranging from no existing infrastructure with minimal resources to dedicated telemedicine IT (information technology) services with bedside devices ready for immediate use and challenges experienced.
COVID-19, telehealth, and pediatric integrated primary care: disparities in service use	Chakawa et al. (14)	The present article explores the variability between in-person pre-COVID and telehealth post-COVID telehealth IPC consultation utilization among children served through a large inner-city primary care clinic. Results suggest that service delivery modality and attendance, referral concerns, and race/ethnicity were significantly associated. Furthermore, the authors reported on differences in attendance rates of children with internalizing vs. externalizing problems and race/ethnicity.
Telephone vs. video visits during COVID-19: Safety-Net provider perspectives	Chang et al. (15)	This article reviews the pros and cons of telephone and video-enabled telemedicine during the first 9 months of the Coronavirus disease 2019 (COVID-19) pandemic as experienced by safety net providers across New York State (NYS). Findings suggest overall positive experiences with telemedicine use by providers, despite challenges.
Addressing pediatric mental health using telehealth during coronavirus disease-2019 and beyond: a narrative review	Cunningham et al. (16)	The present article provides a narrative review of the use of telehealth for mental health care for pediatric populations during the COVID-19 pandemic. Authors reported on a number of themes, including the importance of meeting patients’ needs (e.g., access to technological resources) to optimize success in using telehealth tools and challenges around provider access to support tools for use during telehealth. The article discusses various factors that helped streamline the transition to telehealth which included health insurance companies agreeing to cover such services, relaxed regulations across technological platforms, and access to resources.
Virtual music therapy: developing new approaches to service delivery	Knott and Block (17)	This article describes the coauthors’ three-tiered scaffold model intended to support the program development and deployment of virtual music therapy (VMT) services in response to changes brought about by the COVID-19 pandemic. The model describes an approach to developing VMT services that directs the clinician’s goals of care in formats that are accessible, appropriate, and best meet the patient/client’s needs and abilities. These tiers involve curating resources necessary for music therapy (i.e., using music or social and emotional learning concepts), developing original content of which is developmentally appropriate for clients, and implementing telehealth according to the AMTA (American Music Therapy Association) guidelines.
Connecting during COVID: the application of teleservices in two integrated perinatal settings	Ehmer et al. (18)	This article reviewed care delivery changes at two clinics in response to the COVID-19 pandemic, the HEART program and PROMISE clinic. Authors report on implementation changes including a variety of technology-based services including telephone interventions and support, virtual visits using iPads during medical visits, and video visits that were accessed from patient homes. The PROMISE clinic saw an increase in patient visits, while the HEART program behavioral health visits continued at pre-COVID rates with variations between ethnic groups.
The impact of the Covid-19 related transition to telehealth on visit adherence in mental health care: an interrupted time series study	Eyllon et al. (19)	This study examined the impact of the transition to telemental health delivery on visit adherence for mental health services in an integrated behavioral health department. Monthly visit data for 12,245 patients from January, 2019 to January, 2021 was extracted from the electronic medical record. Telemental health use during the Covid-19 pandemic was associated with improved visit adherence over time, and authors suggest this may be a promising model for improving the efficiency of mental health care delivery after the COVID-19 pandemic.
Virtual care expansion in the Veterans Health Administration during the COVID-19 pandemic: clinical services and patient characteristics associated with utilization	Ferguson et al. (20)	This study described the shift from in-person to virtual care within Veterans Affairs (VA) during the early phase of the COVID-19 pandemic and identified at-risk patient populations who require greater resources to overcome access barriers to virtual care. Veterans with high clinical or social need had higher likelihood of virtual service use early in the COVID-19 pandemic; however, older, homeless, and rural Veterans were less likely to have video visits. For these patients, addressing barriers to accessing care is especially important to reduce disparities in access to treatment.
Implementation of telehealth during COVID-19: implications for providing behavioral health services to pediatric patients	Frye et al. (21)	This study surveyed 25 pediatric behavioral health providers at a single center before and after their first month of utilizing telehealth during coronavirus disease 2019 (COVID-19). Their results indicated the majority of behavioral health providers had no experience providing telehealth services prior to COVID-19, they utilized telehealth to provide pediatric patient care within the first month of access to telehealth, during which reported their confidence in ability use telehealth service delivery significantly improved, regardless of previous training in telehealth. This study identified technological issues as the largest actual barrier to service delivery. Although most participants reported intentions to continue utilizing telehealth when providing behavioral health services for certain types (e.g., diagnostic interviews and outpatient therapy), participants reported a preference for an in-person service delivery. Common reasons endorsed by participants which explain these findings were that it allows for better rapport-building, behavioral observations, reduced technological barriers, and fewer distractions.
The rise of tele-mental health in perinatal settings	Geller et al. (22)	This article reviewed the increase of tele-mental health service delivery in settings who provide care for expectant parents in fetal care centers and parents with children receiving treatment in neonatal intensive care units within a pediatric institution, following the COVID-19 pandemic. This article also included a review of relevant practice regulations, challenges and advantages associated with the change in modality shifting to a tele-mental health delivery system in perinatal settings.
Clinician satisfaction with rapid adoption and implementation of telehealth services during the COVID-19 pandemic	Gentry et al. (23)	This study examined clinician attitudes and perspectives on the acceptability, appropriateness/suitability, and feasibility after changing their service delivery model to use video telehealth to deliver mental health services in response to the COVID-19 pandemic. Overall, mental health clinicians (e.g., psychiatrists, psychologists, trainees such as residents and fellows, advanced practice providers, and licensed mental health counselors) and their patients (79.5%) reported high levels of satisfaction with video telehealth visits. For clinicians these were related to higher levels of acceptability, feasibility, and appropriateness of video telehealth. Moreover, 107 (95.5%) of clinicians responded that they would like video telehealth visits to represent at least 25% of their practice in the future. These findings were explained by positive attitudes about the modality change, and support for the continuation of video telehealth visits as a significant portion of clinical practice.
The impact of COVID-19 on opioid treatment programs in the United States	Goldsamt et al. (24)	This article reviewed the impact of COVID-19 on the delivery of psychopharmacological treatments provided in opioid treatment programs, as well as how public health regulations (e.g., social distancing, stay at home orders) disrupted treatment services offered prior to the pandemic onset. The authors also discussed how clinic directors handled implementing required changes imposed by federal and state regulators through a framework incorporating feedback from staff and patients to better guide feasibility of these changes.
Gender-affirming care without walls: Utilization of telehealth services by transgender and gender diverse people at a federally qualified health center	Grasso et al. (25)	This paper details a telehealth program implemented during the COVID-19 pandemic for transgender and gender diverse (TGD) patients at a federally qualified health center in Boston, Massachusetts. Services were utilized by TGD patients (*n* = 3,189) from 24 U.S. states. During a 6-month period (March–August 2020), Fenway Health cared for close to as many unique TGD patients via telehealth as it did via in-person services during calendar year 2019 (3,794 medical patients in 2019 vs. 3,033 in March through August 2020 [95%]; 946 BH patients in 2019 vs. 911 in March through August 2020 [96%]).
Navigating uncharted waters: Considerations for training clinics in the rapid transition to telepsychology and telesupervision during COVID 19	Hames et al. (26)	This article reviews the shift to telepsychology among North American university training clinics in response to the COVID-19 pandemic and offers relevant suggestions for mental health service providers rapidly transitioning to telepsychology. Results suggest that the majority of university training clinics in North America transitioned to providing services exclusively via telepsychology.
Delivering intensive PTSD treatment virtually: the development of a 2-week intensive cognitive processing therapy-based program in response to COVID-19	Held et al. (27)	This paper details several design considerations as well as patient selection procedures for a 2-week virtual intensive treatment program (vITP) for veterans with posttraumatic stress disorder (PTSD) implemented during the COVID-19 pandemic. In addition, two cases of veterans who successfully completed the vITP are discussed.
Multimodule web-based COVID-19 anxiety and stress resilience training (COAST): Single-cohort feasibility study with first responders	Heyen et al. (28)	This study discusses the implementation of an unguided electronic mental health program, COVID-19 Anxiety and Stress Resilience Training (COAST), tailored to first responders and health care personnel. Findings suggest feasibility and overall satisfaction with the program by first responders.
COVID-19 impact on learning among New York state providers and learners	Hinds et al. (29)	This article reviews provider engagement with training and resources for online content delivery provided by the Center for Practice Innovation (CPI). Results found an increase in new training registrations from March–June 2020, compared to 6 months prior, for both in state and out of state behavioral health providers.
The provision of counseling to patients receiving medications for opioid use disorder: Telehealth innovations and challenges in the age of COVID-19	Hughto et al. (30)	This article discusses the implementation of counseling services via a hybrid telehealth/in-person MOUD treatment model, drawing data from a Rhode Island-based clinic.
Mental health appointments in the era of COVID-19: Experiences of patients and providers	Hunsinger et al. (31)	This study explored the impact of changes in delivery of mental health care services during the pandemic on patient and provider satisfaction with care. Findings indicate provider and patient satisfaction with telehealth visits. Results suggest that telepsychiatry is an acceptable, although not always preferred, modality for psychiatric care during the global pandemic.
What to Do When Being There Means Being Vulnerable	Ihle (32)	This article discusses the implementation of remote consult and liaison services at an inpatient psychiatric hospital during the COVID-19 pandemic.
Changes in access to educational and healthcare services for individuals with intellectual and developmental disabilities during COVID-19 restriction	Jeste et al. (33)	This study describes changes in healthcare access and educational services for individuals with Intellectual and Developmental Disabilities (IDD) that occurred as a result of COVID-19 restrictions. Survey results found that COVID-19 restrictions greatly affected access to services for individuals with syndromic IDDs. Findings indicate that telehealth may provide opportunities for delivery of care and education in a sustainable way.
Conducting CBT for Anxiety in Children with Autism Spectrum Disorder During COVID19 Pandemic	Kalvin et al. (34)	This article describes the transition to remote delivery of cognitive-behavioral therapy (CBT) for anxiety in children with autism spectrum disorder (ASD) who participated in a clinical trial during the COVID-19 pandemic.
COVID-19 Tele-Mental Health: Innovative Use in Rural Behavioral Health and Criminal Justice Settings	Krider and Parker (35)	The authors of this article conducted semi-structured phone interviews with five senior-level professionals involved in tele-MH provision across four rural U.S. regions concerning tele-MH services before and since COVID-19. The article discusses the benefits of the expanded technology, challenges to implementation, and any data collected thus far regarding satisfaction and effectiveness of tele-MH.
Staying Connected In The COVID-19 Pandemic: Telehealth At The Largest Safety-Net System In The United States	Lau et al. (36)	This article discusses care delivery implementation changes during the COVID-19 at the largest safety-net healthcare delivery system in the United States, New York City Health + Hospitals. Findings demonstrate that they were able to transform the system using virtual care platforms through which they conducted almost eighty-three thousand billable televisits in 1 month, as well as more than thirty thousand behavioral health encounters via telephone and video. Telehealth also enabled them to support patient–family communication, post discharge follow-up, and palliative care for patients with COVID-19.
Preserving Continuity of Behavioral Health Clinical Care to Patients Using Mobile Devices	Little et al. (37)	The present article details the use of mobile technology for performing tele-behavioral health visits as a viable option for continued care to warfighters. The research team assessed existing Internet protocol-based desktop teleconferencing solutions, generically known as a Web Real-Time Communications (WebRTC) system, for establishing a secure connection to a Service Members personal mobile device outside of the Department of Defense (DoD) network. Results found that it was technically feasible to provide desktop video tele-conference capabilities from a .mil computer to a personal mobile device without compromising DoD security and information assurance requirements using future WebRTC systems.
“It’s splendid once you grow into it:” Client experiences of relational teletherapy in the era of COVID- 19	Maier et al. (38)	This article explores the lived experiences of individuals in teletherapy, specifically those engaging in teletherapy with a romantic partner or family member. The authors use a thematic analysis of open-ended online survey questions to explore the experiences of 25 individuals who engaged in couple or family teletherapy after social distancing began. Themes that emerged included “making do,” safe therapeutic space, convenience, logistical challenges, and therapist accommodation.
Family-based treatment via videoconference: Clinical recommendations for treatment providers during COVID-19 and beyond	Matheson et al. (39)	This article discusses common challenges clinicians may encounter in providing family-based treatment (FBT) via TeleHealth for children and adolescents with anorexia nervosa and bulimia nervosa. The authors also discuss possible solutions and offer practical considerations for providers delivering FBT in this format.
Telehealth Delivery of a Behavioral Parent Training Program to Spanish-Speaking Latinx Parents of Young Children With Developmental Delay: Applying an Implementation Framework Approach	McIntyre et al. (40)	The authors delivered a behavioral parent training program via telehealth to 42 Spanish-speaking Latinx parents of preschool children with developmental delay (DD) and elevated behavior problems during the pandemic. They used an implementation framework to examine acceptability, adoption, appropriateness, feasibility, fidelity, and implementation cost of the telehealth approach for this sample. The findings suggest overall positive implementation outcomes for Latinx parents of children with DD.
Use of Telehealth in Substance Use Disorder Services During and After COVID-19: Online Survey Study	Molfenter et al. (41)	This study examined the adoption of these technologies across the SUD service continuum, acceptance of these technologies among service providers, and intent of providers to use these technologies after the pandemic. The overall perceived ease of use and usefulness of telephonic and video services suggest promising post–COVID-19 applications of these services. Survey participants consistently preferred video services to telephonic services; however, the availability of telephonic services to those lacking easy access to video technology is an important characteristic of these services.
Increased availability of telehealth mental health and substance abuse treatment for peripartum and postpartum women: A unique opportunity to increase telehealth treatment	Moreland et al. (42)	This article details the adaptation of mental health screenings and treatments to remote platforms at the Medical University of South Carolina’s (MUSC) Women’s Reproductive Behavioral Health Program. Outpatient mental health and substance use treatments were converted to online platforms for peripartum and postpartum women with Opioid Use Disorder (OUD). Results found that the transition to telehealth-based services for women with OUD is promising.
Study of Impact of Telehealth Use on Clinic “No Show” Rates at an Academic Practice	Muppavarapu et al. (43)	The authors examined the clinic no-show rate across different modalities of care delivery (Face to Face, Telephone visits and Audio–Video visits). Using technology in health care delivery can decrease the clinic no show rate. Digital literacy for patients and providers is critical for successful utilization of telehealth.
Home direct-to-consumer telehealth solutions for children with mental health disorders and the impact of Covid-19	Norman et al. (44)	This article discusses a home direct-to-consumer telehealth program initiated in 2016 and utilized during the COVID-19 pandemic. Authors found an increase in the utilization of mental health encounters via telehealth. Findings indicate that telemental health was more effective in meeting targeted volumes than the overall health system.
Chasing the Curve: Program Description of the Geisinger PrimaryCare Behavioral Health Virtual First Response to COVID-19	O’Dell et al. (45)	This article reports on a telebehavioral health (TBH) services delivery model developed by the Geiser rural health system. The authors report outcomes of their Virtual First approach from March 2020 through June 2020, as well as provider surveys of acceptability and usability of the InTouch platform used for TBH. They found that they were able to attain or surpass comparable patient access and utilization by implementing TBH services compared with in-person services over the last 2 years while maintaining a training program.
Impact of the COVID-19 Pandemic on Child and Adolescent Mental Health Policy	Palinkas et al. (46)	The authors conducted a qualitative study to determine the impact of the COVID-19 pandemic on implementation of evidence-based policy and practice by State Mental Health Authorities (SMHA) for prevention and treatment of mental health problems in children and adolescents. Semi-structured interviews were conducted with 29 SMHA of 21 randomly selected states stratified by coronavirus positivity rate and rate of unmet services need. Findings suggest that there was an increased need for services during the pandemic due primarily to family stress and separation from peers. Some of the barriers to telehealth use identified included limited access to internet technology, family preference for face-to-face services, lack of privacy, difficulty using with young children and youth in need of substance use treatment, finding a Health Insurance Portability and Accountability Act (HIPAA)-compliant platform, training providers and clients, and reimbursement challenges.
Efficacy of intensive CBT telehealth for obsessive–compulsive disorder during the COVID-19 pandemic	Pinciotti et al. (47)	This article compares the trajectory and outcomes of two matched samples using linear mixed modeling: a pre-COVID in-person (IP) sample and COVID TH sample. Findings suggested that both modalities are effective at treating OCD and depressive symptoms, although the pandemic TH group required an additional 2.6 treatment days. The current study provides evidence that PHP and IOP treatment delivered via TH during the COVID-19 pandemic is approximately as effective as pre-pandemic IP treatment and provides promising findings for the future that individuals with complicated OCD who do not have access to IP treatment can still experience significant improvement in symptoms through TH PHP and IOP treatment during and potentially after the pandemic.
Feasibility and initial outcomes of a group-based teletherapy psychiatric day program for adults with serious mental illness: Open, nonrandomized trial in the context of COVID-19	Puspitasari et al. (48)	This single-arm, nonrandomized pilot study aimed to assess the feasibility and initial patient-level outcomes of a psychiatric transitional day program that switched from an in-person group to a video teletherapy group during the COVID-19 pandemic. All patient-level reported outcomes demonstrated significant improvements in depression, anxiety, overall suicide risk, wish to live, wish to die, and overall mental health from admission to discharge. Rapid adoption and implementation of a group-based teletherapy day program for adults at risk of psychiatric hospitalization appeared to be feasible and effective. Patients demonstrated high completion and attendance rates and reported significant improvements in psychosocial outcomes.
Rapid adoption and implementation of telehealth group psychotherapy during COVID 19: Practical strategies and recommendations	Puspitasari et al. (49)	In this paper, the authors describe their approach to quickly adapting to a teletherapy technology platform for an intensive outpatient program (IOP) guided by cognitive and behavioral modular principles for adults with serious mental illness. A review of existing guidelines was conducted and the staged approach for teletherapy implementation was selected as the most appropriate model for their organizational context. This model of rapid teletherapy implementation offers practical clinical guidelines for administrators and clinicians seeking to transition traditional in-person behavioral health services to a teletherapy format.
Case studies from the digital clinic: integrating digital phenotyping and clinical practice into today’s world	Rauseo-Ricupero et al. (50)	This article is a case series which provides several examples from the Digital Clinic, an outpatient mental health program which uses smartphone technology to augment traditional mental health care. The themes highlighted in this piece, expanding emotional-awareness, symptom tracking, and medication management, provide real-clinical examples of how the Digital Clinic offered remote mental health care to a diverse group of people. Furthermore, the article demonstrates to practicing clinicians how digital technologies, like smartphone apps, can diversify methods of clinical engagement, assist with collecting health metrics in a safe and ethical manner, and promote person centered care.
Virtual mental health care in the veterans’ health administration’s immediate response to coronavirus disease-19	Rosen et al. (51)	This article discusses research on the effectiveness of telemental health, VHA policies before COVID-19 that facilitated the use of telemental health systemwide, and VHA’s actions that rapidly scaled use of telemental health during the first months of the outbreak. Key challenges and lessons learned from VHA’s experience and implications for providers and health care systems regarding the use of telemental health to meet patients’ mental health care needs during the pandemic are also discussed.
Breaking down barriers: Young adult interest and use of telehealth for behavioral health services	Rosenthal et al. (52)	This study examines which young adults in Rhode Island were using these telehealth services and are interested in its use. Results suggest sexual and gender minorities and those with low social status were more likely to access these services, highlighting its effectiveness at reaching disadvantaged young adults. Those with mental health symptoms were more likely to utilize telehealth, but those with substance use were not. Continued coverage and use of telehealth for mental health and substance use services is essential in breaking down barriers to care for young adults in Rhode Island.
Effective and accessible telephone-based psychotherapy and supervision	Rowen et al. (53)	The focus of this paper was to examine the effectiveness of psychotherapy treatment and clinical supervision, via telephone delivery (i.e., audio-only calls), in a university training clinic, which primarily serves a low-income, community population. Participants were ethnically diverse, adult, outpatient psychotherapy clients from a large, urban setting and the university community. Clinician report of supervision alliance (SWAI-T), satisfaction, and other supervisory variables during remote supervision were comparable to in-person supervision and reflected good alliance and satisfaction for both modalities. These data suggest that use of audio-only calls for supervision and treatment provision can be effective for training and care, especially when clients have established therapeutic relationships with their clinicians.
COVID-19 and music therapists’ employment, service delivery, perceived stress, and hope: A descriptive study	Gaddy et al. (54)	The purpose of this study was to determine the impact of the pandemic on the employment, service delivery, stress, and hope of music therapy professionals in the United States. Music therapists (N = 1,196) responded to a 51-item survey including questions related to employment and service delivery. The study also included the Adult Hope Scale and the Perceived Stress Scale-10 (PSS-10). Results indicated that many music therapists experienced changes in their positions, including a decrease in client contact hours and an increase in using alternative services, such as telehealth. Changes in service hours and delivery were higher for individuals who worked in private practice than for other settings. Primary respondent concerns included being a carrier of COVID-19, being isolated from loved ones, and income loss.
Telebehavioral health during the COVID-19 pandemic: A qualitative analysis of provider experiences and perspectives	Schoebel et al. (55)	This qualitative study describes behavioral health provider perspectives on the use of telebehavioral health before and during the pandemic and how policy changes impacted access to and utilization of behavioral health services in Michigan. The thematic analysis resulted in four overarching themes: (1) increased access to care; (2) maintenance of quality of care; (3) minimal privacy concerns; and (4) client and provider satisfaction. During and post-pandemic, providers need flexibility to determine whether in-person or telebehavioral health services, including audio-only, best meet client needs. Providers identified several populations for which telebehavioral health was less accessible: clients with serious mental illness and substance use disorder, those with no broadband Internet access, children, and older adults. Additional training in telebehavioral health service provision can positively impact quality of care.
Implementation of home-based telemental health in a large child psychiatry department during the COVID-19 crisis	Sharma et al. (56)	The authors present the implementation of a home-based TMH (HB-TMH) service during the COVID-19 pandemic. The transition was accomplished in 6 weeks. Most in-clinic services were rapidly moved off campus to the home. Owing to challenges encountered with each implementation component, phone sessions bridged the transition from in-clinic to reliable virtual appointments. Within 3 weeks (March 20, 2020) of planning for HB-TMH, 67% of all appointments were conducted at home, and within 4 weeks (March 27, 2020), 90% were conducted at home. By week 6 (April 3, 2020), reliable HB-TMH appointments were implemented.
Let us face it: video conferencing psychotherapy requires the extensive use of ostensive cues	Fisher et al. (57)	This paper provides a brief background on the concept of epistemic trust, research on the determinants of its development and the integrative framework it provides for some traditional concepts in psychotherapy, such as the therapeutic alliance. In particular, research on ostensive cuing, which requires significant modification in a remote delivery context, has important implications in the transition to VCP. This knowledge may be of value to psychotherapists, who are required to make substantial changes in the nature of the encounter with their patients, and may help them identify benefits and hindrances that might arise from this transition, as well as pointing out techniques that may encourage effective adaptation to the change.
Navigating changes in the physical and psychological spaces of psychotherapists during Covid-19: When home becomes the office	Shklarski et al. (58)	This article reports on a mixed-method study focused on assessing psychotherapists’ most significant challenges and specific adaptations to this experience of providing remote therapy from home during the Covid-19 pandemic. Findings from the study revealed that the shared trauma experienced as a result of the pandemic, the unexpected and sudden transition to the new therapeutic setting, and “Zoom fatigue” were among the most significant challenges faced by therapists. The participants also demonstrated great resilience as they found creative ways to adapt and continue their meaningful work with their clients. This was especially true for those working with children. Ultimately, the participants had mixed feelings about the possibility of returning to the office setting.
Assessing the impact of COVID-19 on mental health providers in the southeastern United States	Slone et al. (59)	The authors conducted a web-based survey of 500 licensed mental health providers to assess their employment and caseloads, logistics of care, quality of care, and patient-provider relationships and communication during the pandemic. Over 90% of providers reported changes to their employment (e.g., furloughs), with 64% no longer practicing. Providers who reported no longer practicing were older in age, racial minorities, served rural communities, worked in small clinics/provider networks, were social workers and marriage and family therapists, and relied on private insurance or out-of-pocket payment. Most practicing providers reported similar-to-increased caseloads (62%), new patients seeking services (67%), and appointment frequency (70%). Approximately 97% of providers used telemedicine, with 54% providing services mostly-to-exclusively via telemedicine. Most providers reported losing contact with patients deemed unstable (76%) or a danger to themselves/others (71%). Most providers reported maintained-to-improved quality of care (83%), patient-provider relationships (80%), and communication (80%). Results highlight concerns relating to mental health services during the pandemic, however practicing providers have demonstrated resilience to coordinate and provide high quality care.
Reflections on changing times.	Stancin (60)	This commentary provides are flection on the early response of 1 established IPC program to the COVID-19pandemic. Results: Initial markers of successful adaptation to care delivery and training models include efforts that incorporate virtual consultation and telehealth practices. Conclusions: The impact of the COVID-19 pandemic on pediatric psychology in IPC settings will largely depend on the flexibility and adaptability of workflows and training methods to meet the needs of the changing landscape.
Patients’ perceptions of telehealth services for outpatient treatment of substance use disorders during the COVID-19 pandemic	Sugarman et al. (61)	This study examined patient perceptions of telehealth in an outpatient SUD treatment program offering individual therapy, group therapy, and medication management. Telehealth visits were a satisfactory treatment modality for most respondents receiving outpatient SUD care, especially those engaging in individual therapy. Challenges remain for telehealth group therapy.
Rapid creation of child telemental health services during COVID-19 to promote continued care for underserved children and families	Tolou-Shams et al. (62)	The COVID-19 pandemic prompted the rapid transformation of child mental health services from mostly in-person to fully remote delivery at an urban safety-net hospital. No-show rates substantially declined when implementing video visits, and the volume of service delivery was unchanged compared to pre-pandemic in-person visits. In addition, no-show rates for telehealth sessions did not increase over time. Recommendations for telehealth quality assurance and improvement to best respond to children and families with existing mental health needs and limited resources during disasters and in their aftermath are suggested.
Perspectives of opioid use disorder treatment providers during COVID-19: Adapting to flexibilities and sustaining reforms	Treitler et al. (63)	This study aimed to understand changes in treatment providers’ care during COVID-19, provider experiences with the adaptations, and perceptions of which changes should be sustained long-term. Findings support sustaining temporary regulatory and payment changes to MOUD practice, which may have improved treatment access and allowed for more flexible, individually tailored patient care. Few negative, unintended consequences were reported by providers, but more research is needed to evaluate the patient experience with changes to practice during the COVID-19 pandemic.
Telehealth use among safety-net organizations in California during the COVID-19 pandemic	Uscher-Pines et al. (64)	The authors described trends in the use of in-person, telephone, and video visits among California FQHCs before and during the COVID-19 pandemic. CMS estimated that 30% of telehealth visits were audio only during the pandemic.Estimates reported here may be higher because low-income patients face unique barriers to accessing video visits and FQHCs lack resources to develop the necessary infrastructure. Study limitations include that only FQHCs in 1 state were tracked. Also, 5 FQHCs reported early challenges distinguishing visit types and categorized all telehealth visits by the dominant modality.
Suddenly becoming a “Virtual Doctor”: Experiences of psychiatrists transitioning to telemedicine during the COVID-19 pandemic	Uscher-Pines et al. (65)	This qualitative study sought to understand how this dramatic change in delivery has affected mental health care, including modes of telemedicine psychiatrists used, barriers encountered, and future plans. The aim was to inform the ongoing COVID-19 response and pass on lessons learned to psychiatrists who are starting to offer telemedicine. Findings highlight that although psychiatrists expressed some concerns about the quality of these encounters, the transition has been largely positive for both patients and physicians.
Cognitive behavioral therapy in the time of the coronavirus	Waller et al. (66)	This article draws together clinician experiences of the issues that should be attended to, and how to address them within a telehealth framework. A range of themes emerged from the online discussion. A large proportion were general clinical and practical domains (patient and therapist concerns about telehealth; technical issues in implementing telehealth; changes in the environment), but there were also specific considerations and clinical recommendations about the delivery of CBT-ED methods.
Group teletherapy for first-episode psychosis: Piloting its integration with coordinated specialty care during the COVID-19 pandemic	Wood et al. (67)	This article describes efforts to implement group teletherapy for two small cohorts of individuals with FEP receiving care in a coordinated specialty care clinic using methods adopted from Acceptance and Commitment Therapy. The authors observed high adherence with group visits as well as client satisfaction across groups. Their experience provides guidance and a model for integration of virtual group therapy within coordinated specialty care (CSC).
Implementing a low-threshold audio-only telehealth model for medication-assisted treatment of opioid use disorder at a community-based non-profit organization in Washington, D.C.	Yeo et al. (68)	This case study describes a transition from a low-threshold community-based in-person MAT clinic to an audio-only telehealth model. The authors found that an audio-only telehealth model was viable. Although they had decreased retention rates following the transition to an audio-only telehealth model, their rates remained excellent compared to published values for in-person MAT care.
Patient preferences for patient portal–based telepsychiatry in a safety net hospital setting during COVID-19: Cross-sectional study	Yue et al. (69)	The authors assessed patient preference for patient portal–based video visits or telephone-only visits and to identify the demographic variables associated with their preference. Among behavioral health patients at a safety net hospital, there was relatively low engagement with video-based visits through the hospital’s patient portal, particularly among older adults.
Patient satisfaction with partial hospital telehealth treatment during the COVID-19 pandemic: Comparison to in-person treatment	Zimmerman et al. (70)	The authors compared patient satisfaction of partial hospital services delivered via telehealth to in-person treatment provided to patients treated prior to the COVID-19 outbreak. The sample included 240 patients who were treated virtually from May, 2020 to October, 2020, and a comparison group of 240 patients who were treated in the in-person partial program a year earlier. Patient satisfaction was as high with telehealth partial hospital treatment as with in-person treatment.

### 3.1 Telehealth

The majority of articles in this review (63%) used the term telehealth to discuss care modality changes implemented during the pandemic. There were slight variations in how authors defined telehealth, with some providing no explicit definition for the term while others provided detailed definitions. For the articles which did not provide a definition of telehealth, they instead offered examples of how telehealth was operationalized in their study (e.g., “telephone, video, email”). Alternatively, some articles broadly defined telehealth to include a range of communication platforms utilizing telephone or videoconferencing (17, 68). Other studies defined telehealth as “remote, real-time (i.e., synchronous) delivery of videoconferencing” or “psychotherapy provided via telephone or videoconferencing platforms,” respectively (34, 54). Some articles were more specific in their definitions, which reflected how they operationalized the term in relation to the change in modality being discussed. For example, one article defined telehealth as “providing clinician-led Family-Based Therapy to patients at a remote distance with the use of a videoconferencing platform” (39). Telehealth encompassed both psychiatry and psychology practice changes.

### 3.2 Telemedicine

Across the articles included in this review, approximately 23% referred to technology-based modality changes such as telemedicine. Telemedicine was identified as a subset of telehealth and defined as the use of telephone or video-enabled technologies to deliver healthcare services when distance is a barrier (16). Telemedicine can be conducted synchronously (e.g., live, real-time audio or audiovisual meetings) and asynchronously (e.g., electronic messaging, patient monitoring), and it can include hybrid formats, such as in-person virtual rounding in an academic hospital setting (17). Telemedicine was not exclusive to a particular provider or service type and was utilized across various settings and populations such as primary care, pediatrics, psychiatry, psychology, neuropsychology, and social work. It was interchangeably used with broader terms such as telehealth.

### 3.3 Telemental health

Approximately 17% of articles in this review used the term telemental health to describe care modality changes implemented during the COVID-19 pandemic. The definition for telemental health broadly included the use of smartphone and/or videoconferencing technologies to deliver mental health services. Some definitions specified whether services were delivered synchronously and/or asynchronously and explicitly defined how services were delivered remotely (e.g., on-site vs. off-site). Telemental health was interchangeably used with telebehavioral health, telepsychiatry, telepsychology, and teletherapy (18). Unlike broader terms such as telehealth and telemedicine, telemental health specifically referred to the delivery of *mental health services* via technology-based modalities.

### 3.4 Telepsychiatry

Approximately 11% of the articles utilized the term telepsychiatry to primarily describe psychiatry consultation-liaison services conducted virtually either by telephone or video in a hospital setting. The modality of telepsychiatry was utilized to provide services to various departments of the hospital, including intensive care units, specialty outpatient clinics, and emergency departments. Care was delivered in hybrid formats, with some services delivered in-person through virtual means (19). Telepsychiatry was interchangeably used with telehealth, telemental health, telemedicine, and teleconsultation ((17, 20, 21)).

### 3.5 Teletherapy

About 9% of the articles in this review utilized the term teletherapy to exclusively describe psychotherapy mental health services provided via virtual methods and platforms, either by telephone or video. Teletherapy was not used in conjunction with other terms. Teletherapy involved the delivery of services across various patient populations, service types (e.g., individual or group therapy), and treatment modalities (e.g., Cognitive Processing Therapy for PTSD) through virtual platforms. Services included synchronous and asynchronous formats.

### 3.6 Telebehavioral health

Telebehavioral health was used in approximately 6% of articles in this review. Similar to telemental health, telebehavioral health was used specifically to describe *mental health* service delivery via virtual/remote platforms. Authors varied in how they defined telebehavioral health. Some articles discussed the format type (hybrid – virtual and in-person care vs. virtual only) in their definition. Most articles defined telebehavioral health to include the use of mobile technology, video conferencing, and audio-only services. Notably, the term telebehavioral health was used in conjunction with other terms in half of the instances, particularly telehealth.

### 3.7 Telepsychology, virtual delivery, videoconferencing psychotherapy, telephone-based psychotherapy, teleconsultation, video telehealth, digital health, unguided mental health program

The remaining modality terms were used only once (Telepsychology, Virtual Delivery, Videoconferencing Psychotherapy, Telephone-based Psychotherapy, Teleconsultation, Video Telehealth, Digital Health, Unguided Mental Health Program). These modality terms were generally used in conjunction with other more widely used terms to describe the mode of delivery of behavioral health services during the pandemic.

In terms of specific delivery formats, care was delivered either synchronously (in real time) or asynchronously. Among the synchronous modalities, patients were engaging in real time with licensed professionals using either audio-only devices (Teleconsultation and Telephone-based psychotherapy) or some form of audio-video conferencing software where they could be seen and heard (telepsychology, virtual delivery, videoconferencing and video telehealth).

The asynchronous deliveries involved the use of self-paced, web-based programs that could be accessed via online applications. The Unguided Mental Health program involved a strictly online or web-based program that could be completed anonymously. This modality was completely self-paced and asynchronous with no guidance from a licensed behavioral health professional (22). Digital Health involved the use of smartphone “app-based support” which followed in-person therapy. The online delivery involved a peer-support model and there were educational components to the app as well (23).

Overall, the common factors seen across terms and definitions included mode of delivery (e.g., telephone and/or videoconferencing technologies) and format type of that service delivery (synchronous vs. asynchronous). Some terms were used as umbrella terms to encompass a wide range of services (e.g., telehealth and telemedicine), while others were specific to a service type (e.g., teletherapy, telepsychology). Variations in definitions were seen in how each term was operationalized. For example, Beran and Sowa (9) defined telepsychiatry to include a “telepresenter” who is responsible for delivering a tablet to the patient to conduct their virtual visit with the doctor. Other definitions of telepsychiatry only specified whether services were offered in-real time or through asynchronous formats (e.g., patient portal videos). Altogether, the results indicate relative agreement between terms and definitions, with slight differences in the application of technology-based services.

## 4 Discussion

The present review identified behavioral healthcare modality changes implemented in the U.S. during March 2020–May 2022 of the COVID-19 pandemic. The pandemic created an opportunity to deliver mental health services virtually on a broader scale than was available pre-pandemic, and the utilization rate of these services has remained higher than pre-pandemic levels (71).

As can be seen in the present review, behavioral or mental health modality changes have been referred to by 14 terms in the literature during the COVID-19 pandemic. Our results show that the most common terms used were telehealth and telemedicine. Given that telehealth and telemedicine are both general terms that refer to different forms of service delivery beyond behavioral and mental health services, moving forward, practitioners and researchers working in the behavioral and mental health care space may wish to retain broad language (e.g., telehealth) while connecting it to more specific language that includes mental or behavioral health (e.g., telemental health). The addition of more specific language can allow practitioners, researchers, and mental health care consumers to identify appropriate literature more readily in evaluating whether a particular form of virtual mental health care is appropriate or effective for a given issue or problem.

On a related point, given the explosion of virtual behavioral and mental health care modalities during and after the COVID-19 pandemic, practitioners and researchers would benefit from standardizing behavioral and mental health care language rather than using a diversity of terminology to refer to the same services. For example, we found that terms such as telemental health, telepsychiatry, and telepsychology were used interchangeably in the literature. This raises the question of why they are all in use. One potential reason for variability in terminology includes the variety of fields involved in mental health care (e.g., counseling, psychology, psychiatry, social work) and the lack of connection between the literatures in these fields. Another potential reason for variability in terminology may be the fact that these services proliferated during COVID-19 among practitioners and in settings that did not commonly use these services. A lack of researcher and practitioner familiarity with the literature and relevant terminology may have resulted in a wide variety and lack of specificity in the terminology being used. Being clearer and more precise in the similarities and differences in terminology could focus the literature regarding these services. A more focused literature can advance efficacy and effectiveness research, practitioner and client access to the literature, and policy advocacy for the services that work.

Our review includes a broad range of organizations and settings that adopted telehealth modality changes during COVID, including state and regional hospitals, pediatric clinics, fetal and neonatal care settings, criminal justice settings, university training clinics, and the Veteran’s Health Administration. Populations served include all ages from young children to older adults, individual, group, family, and couple modalities, and a full range of psychological issues and diagnoses.

The increased use of telehealth during COVID-19 for mental and behavioral healthcare issues has shown that telehealth is feasible and can reduce barriers to care. Telehealth can increase accessibility to mental health services moving beyond the pandemic, which will require ongoing training and development of clear guidelines (72). The appropriate use of telehealth requires specific knowledge related to issues such as privacy protection. For example, awareness of requirements that virtual platforms must have to ensure that protected health information is secure is critical to the sustainability of telehealth (69).

Barriers to using telehealth for behavioral and mental health have included issues around insurance reimbursement policies (73), privacy concerns associated with virtual delivery (e.g., a secure platform), virtual platform accessibility, particularly in rural areas with less internet access (74), concerns about the efficacy of telehealth services (3) or a provider preference for face-to-face services (75) despite evidence indicating that some clients prefer virtual delivery and that behavioral and mental health services provided virtually are generally effective [e.g., (76)].

To address the opportunities and barriers to telehealth for behavioral and mental health care, sustainable virtual mental healthcare options will require policy changes and support. For example, the previous and present U.S. administrations have supported access to virtual mental health services during COVID. The current White House advocates for continued insurance coverage for telemental health and to support delivery across state lines. In addition, recent legislation has extended Medicare telehealth coverage until December 31, 2024, underscoring the importance of these services.

Evidence to date suggests that virtual mental healthcare options are effective and reduce barriers to care, making sustainability of this modality of treatment a high priority (77). The present review provides evidence of how a wide variety of people with a wide range of issues have been supported by virtual mental healthcare options during the COVID-19 pandemic. We urge researchers and practitioners to continue to use, investigate, refine, and promote forms of telehealth treatment that work in ensuring accessibility of mental health care options for all.

## Author contributions

KE: Data curation, Formal analysis, Methodology, Project administration, Writing – original draft, Writing – review & editing. EN: Formal analysis, Methodology, Writing – original draft, Writing – review & editing. NS-R: Formal analysis, Writing – original draft, Writing – review & editing. VD: Formal analysis, Methodology, Writing – original draft, Investigation, review & editing. EB: Formal analysis, Writing – original draft, review & editing. NY: Formal analysis, Writing – original draft. MM: Formal analysis, Writing – original draft. SS: Formal analysis, Writing – original draft. BK: Writing – review & editing. KS: Writing – review & editing.
